# Assessing the effect of oral activated vitamin D on overall survival in hemodialysis patients: a landmark analysis

**DOI:** 10.1186/s12882-018-1111-2

**Published:** 2018-11-06

**Authors:** Jo-Yen Chao, Hsu-Chih Chien, Te-Hui Kuo, Yu-Tzu Chang, Chung-Yi Li, Ming-Cheng Wang, Yea-Huei Kao Yang

**Affiliations:** 1 Division of Nephrology, Department of Internal Medicine, National Cheng Kung University Hospital, College of Medicine, National Cheng Kung University, No.1, University Road, Tainan, 70101 Taiwan; 2 Institute of Clinical Pharmacy and Pharmaceutical Sciences, College of Medicine, National Cheng Kung University, Tainan, Taiwan; 3 Department of Public Health, College of Medicine, National Cheng Kung University, Tainan, Taiwan; 4 Graduate Institute of Clinical Medicine, College of Medicine, National Cheng Kung University, Tainan, Taiwan

**Keywords:** Hemodialysis, Activated vitamin D, Prescribing pattern, Mortality, End-stage renal disease (ESRD)

## Abstract

**Background:**

Patients with end stage renal disease have a high all-cause and cardiovascular mortality. Secondary hyperparathyroidism and vitamin D deficiency are considered part of the mechanism for the excess mortality observed. We aimed to evaluate the relationship between vitamin D use and all-cause mortality.

**Methods:**

In this retrospective cohort study, we included all incident patients who started hemodialysis in Taiwan between 2001 and 2009. Patients were followed from landmark time, i.e., the 360th day from hemodialysis initiation, through the end of 2010 or death. We evaluated the association between activated vitamin D use or not before landmark time and all-cause mortality using conditional landmark analysis with Cox regression. We used group-based trajectory model to categorize high-dose versus average-dose users to evaluate dose-response relationships.

**Results:**

During the median follow-up of 1019 days from landmark time, vitamin D users had a lower crude mortality rate than non-users (8.98 versus 12.93 per 100 person-years). Compared with non-users, vitamin D users was associated with a lower risk of death in multivariate Cox model (HR 0.91 [95% CI, 0.87–0.95]) and after propensity score matching (HR 0.94 [95% CI, 0.90–0.98]). High-dose vitamin D users had a lower risk of death than conventional-dose users, HR 0.75 [95% CI, 0.63–0.89]. The association of vitamin D treatment with reduced mortality did not alter when we re-defined landmark time as the 180th day or repeated analyses in patients who underwent hemodialysis in the hospital setting.

**Conclusions:**

Our findings supported the survival benefits of activated vitamin D among incident hemodialysis patients.

**Electronic supplementary material:**

The online version of this article (10.1186/s12882-018-1111-2) contains supplementary material, which is available to authorized users.

## Background

Cardiovascular disease is an important cause of death in patients with chronic kidney disease (CKD) [1, 2]. Apart from diabetes, dyslipidemia, and atherosclerosis, non-traditional risk factors, especially secondary hyperparathyroidism, vascular calcification, and heart failure, all play important roles in patients with CKD and end stage renal disease (ESRD) [3–6]. In addition, vitamin D insufficiency and deficiency, which result from malnutrition, reduced 1α-hydroxylase activity, and increased fibroblast growth factor-23, are highly prevalent in advanced CKD and contribute to secondary hyperparathyroidism and adverse cardiovascular outcomes [7].

In the literature, low 25-hydroxyvitamin D and 1, 25-dihydroxyvitamin D levels are associated with increased all-cause and cardiovascular mortality in the general population, CKD, and uremic patients [8–14]. Pleiotropic effects of activated vitamin D include improving endothelial function, inhibition of vascular smooth muscle proliferation and vascular calcification, suppression of renin production, and modification of inflammatory response [15–18]. Treatment with activated vitamin D is associated with lower incidence of left ventricular hypertrophy, myocardial fibrosis, and pulmonary congestion [17, 19, 20].

Findings from observational studies have suggested that administration of activated vitamin D was associated with reduced mortality and improved cardiovascular outcome in advanced CKD and ESRD patients [21–25]. Results from one study had ever suggested that patients treated with oral activated vitamin D had a 45% reduction in mortality but the survival benefit was inversely related to the vitamin D dose [22]. Findings from another meta-analysis of randomized controlled trials had suggested that treatment of vitamin D compounds was associated with increased risk of hypercalcemia and hyperphosphatemia while inconsistently reducing parathyroid hormone (PTH) levels. The potential beneficial effect on mortality was unproven and underpowered to be evaluated because only few studies reported clinical hard outcomes [26].

In clinical practice, concerns about hypercalcemia and potential vascular calcification have confined treatment of vitamin D in patients with elevated PTH and with relatively low calcium levels. Besides, patients prescribed vitamin D are generally younger and healthier, implying unmeasured confounders that could not be removed by statistical adjustment, which could have biased the findings from previous studies [22, 27, 28].

In Taiwan, the prevalence of ESRD reached 2584 per million in 2010, while rates of 2260 and 1870 were reported in Japan and the United States [29]. Given the potential benefits of activated vitamin D mentioned above, we hypothesized that prescription of activated vitamin D should improve overall outcome in ESRD patients. Regarding the universal coverage of health care and bundled payment for dialysis in Taiwan, the National Health Insurance Research Database (NHIRD) can be employed to examine the effect of activated vitamin D in the real world setting and establish the domestic evidence for clinical practice.

Using NHIRD, we aimed to determine the prevalence of activated vitamin D prescriptions, including calcitriol and alfacalcidol, in incident hemodialysis patients in Taiwan and the association of vitamin D use with potential effect on all-cause mortality.

## Methods

### Data sources

Taiwan National Health Insurance (NHI) provides comprehensive health care service to over 23 million residents, covering more than 99% of the population in Taiwan since 1995. The NHIRD is established from the de-identified claims data of NHI, which comprise demographic data of enrollees, information of healthcare professionals, medical facilities, and service claims from ambulatory care, hospital admission, and contracted pharmacies.

The registry of catastrophic illness patients is a subset of NHIRD that covers patients with specific severe disease conditions that require close and costly medical care. Because patients with catastrophic illness certificate (CIC) are exempted from co-payment for related medical services, this registry is representative of most, if not all, patients with medically qualified diseases. In Taiwan, ESRD patients with uremia and dialysis dependence are eligible for CIC when they initiate maintenance dialysis, which is reviewed and approved by nephrologists in the National Health Insurance Administration.

All diagnoses in the NHIRD were coded according to the International Classification of Disease, 9th revision, Clinical Modification (ICD-9-CM).

### Study design, population and outcome

We included all incident uremic patients that initiated hemodialysis between January 1, 2001 and June 30, 2009. Patients who were younger than 20 years or had past history of malignancy were excluded. Those who had kidney transplant graft failure and re-initiated dialysis were also excluded due to a very small number of patients and different patient characteristics regarding chronic kidney disease and mineral bone disorders. The diagnosis of uremia and long-term dialysis dependence was confirmed using the database of catastrophic illnesses.

The date of the first hemodialysis treatment was defined as the cohort entry date. Concerning that hemodialysis patients had a highest mortality rate during the first year following dialysis initiation [30], we applied landmark design and patients were followed from the 360th day after cohort entry until death or the end of 2010. The study protocol was approved by the Institutional Review Board (IRB) of National Cheng Kung University Hospital (IRB number: A-EX-104-037).

### Baseline information and covariates

Baseline information including age, sex, vascular access type, baseline comorbidities, and medications were showed in Table 1. Information of baseline comorbidities were retrieved using diagnostic codes from the claims data of ambulatory care or hospital admission within 90 days prior to or after the date of cohort entry, i.e. the baseline period. We applied the diagnostic codes modified from the Elixhauser comorbidity index to define comorbidities (Additional file 1: Appendix S1) [31]. Co-medications including antiplatelets, warfarin, anti-diabetic agents, statins, angiotensin-converting enzyme inhibitors/Angiotensin II receptor blockers, beta-blockers, diuretics, and erythropoiesis-stimulating agents (Additional file 1: Appendix S2) were retrieved as well during the baseline period. Information of vascular access type (Additional file 1: Appendix S3) were retrieved using procedure codes from claims data of ambulatory care or hospital admission within 360 days prior to or 180 days after the hemodialysis initiation.

**Table 1:** Baseline characteristics of activated vitamin D users versus non-users according to status by landmark time, before and after propensity score (PS) matching

	Entire cohort			After PS match		
	Vitamin D users	Non-users	*d* ^a^	Vitamin D users	Non-users	*d* ^a^
N (%)	8151 (15.5)	44,606 (84.5)		7232 (25.0)	21,696 (75.0)	
Age, year	58.9 (14.1)	62.5 (13.3)	0.26	60.7 (13.5)	60.8 (13.7)	< 0.01
< 53	2847 (34.9)	10,949 (24.6)	0.25	1933 (26.7)	5967 (27.5)	0.02
≥ 53 and < 64	2128 (26.1)	11,653 (26.1)		1932 (26.7)	5728 (26.4)	
≥ 64 and < 73	1749 (21.5)	11,325 (25.4)		1776 (24.6)	5123 (23.6)	
≥ 73	1427 (17.5)	10,679 (23.9)		1591 (22.0)	4878 (22.5)	
Gender (male)	3680 (45.2)	22,619 (50.7)	0.11	3540 (48.9)	10,647 (49.7)	< 0.01
Comorbidities						
DM	3327 (40.8)	26,616 (59.7)	0.38	3325 (46.0)	10,032 (46.2)	< 0.01
CHF	2200 (27.0)	15,195 (34.1)	0.15	2136 (29.5)	6438 (29.7)	< 0.01
MI	1932 (23.7)	13,868 (31.1)	0.17	1896 (26.2)	5574 (25.7)	0.01
PVD	259 (3.2)	1509 (3.4)	0.01	242 (3.4)	687 (3.2)	0.01
CVD	774 (9.5)	7095 (15.9)	0.19	774 (10.7)	2388 (11.0)	0.01
COPD	14 (0.2)	128 (0.3)	0.02	14 (0.2)	40 (0.2)	< 0.01
CTD	176 (2.2)	1021 (2.3)	< 0.01	163 (2.3)	498 (2.3)	< 0.01
PUD	1344 (16.5)	8023 (18.0)	0.04	1252 (17.3)	3605 (16.6)	0.02
Neoplasia	10 (0.1)	56 (0.1)	< 0.01	9 (0.1)	30 (0.1)	< 0.01
Chronic liver diseases	1001 (12.3)	5353 (12.0)	< 0.01	917 (12.7)	2643 (12.2)	0.02
Vascular access type			0.15			0.06
AVF	6372 (78.2)	34,240 (76.7)		5811 (80.4)	17,145 (79.2)	
AVG	617 (7.6)	4308 (9.7)		585 (8.1)	1833 (8.5)	
Permanent catheter	116 (1.4)	1097 (2.5)		110 (1.5)	443 (2.0)	
Double lumen catheter	539 (6.6)	3219 (7.2)		389 (5.4)	1392 (6.4)	
Unknown	507 (6.2)	1742 (3.9)		337 (4.7)	883 (4.1)	
Medications						
Antiplatelets^b^	3929 (48.2)	24,796 (55.6)	0.15	3687 (50.9)	10,900 (50.2)	0.01
Aspirin / Clopidogrel	2324 (28.5)	15,600 (35.0)	0.14	2189 (30.3)	6567 (30.3)	< 0.01
Cilostazol	154 (1.9)	1146 (2.6)	0.05	147 (2.0)	440 (2.0)	< 0.01
Warfarin	143 (1.8)	988 (2.2)	0.03	140 (1.9)	387 (1.8)	0.01
Statins	1373 (16.8)	9535 (21.4)	0.12	1303 (18.0)	3820 (17.6)	0.01
Insulin	1615 (19.8)	12,898 (28.9)	0.21	1604 (22.2)	4825 (22.2)	< 0.01
OAD	1812 (22.2)	16,003 (35.9)	0.30	1809 (25.0)	5579 (25.7)	0.02
Metformin	179 (2.2)	1857 (4.2)	0.11	179 (2.5)	530 (2.4)	< 0.01
Sulfonylurea	917 (11.3)	8041 (18.0)	0.19	917 (12.7)	2893 (13.3)	0.02
α-glucosidase inhibitors	148 (1.8)	1478 (3.3)	0.09	148 (2.1)	458 (2.1)	< 0.01
TZD	81 (1.0)	684 (1.5)	0.05	80 (1.1)	253 (1.2)	< 0.01
DPP-4 inhibitors	2 (0.02)	5 (0.01)	0.01	0 (0.00)	1 (0.00)	< 0.01
Meglitinides	485 (6.0)	3938 (8.8)	0.11	485 (6.7)	1444 (6.7)	< 0.01
ACEI / ARB	3972 (48.7)	23,726 (53.2)	0.09	3569 (49.4)	10,730 (49.5)	< 0.01
Beta-blockers	4173 (51.2)	24,243 (54.4)	0.06	3764 (52.1)	11,205 (51.7)	0.01
Diuretics	5737 (70.4)	34,377 (77.1)	0.15	5229 (72.3)	15,793 (72.8)	0.01
ESA	1887 (23.2)	10,133 (22.7)	0.01	1691 (23.4)	5011 (23.1)	0.01

*Note*:

(1) The landmark time is the 360th day of initiation of hemodialysis

(2) Values for categorical variables are given as numbers (percent); for continuous variables, as means (standard deviation)

*Abbreviations*: *DM* diabetes mellitus, *CHF* congestive heart failure, *MI* myocardial infarction, *PVD* peripheral vascular disease, *CVD* cerebrovascular disease, *COPD* chronic obstructive pulmonary disease, *CTD* connective tissue disease including rheumatoid arthritis, systemic lupus erythematosus, etc, *PUD* peptic ulcer disease; Chronic liver diseases: chronic viral hepatitis, cirrhosis and its complications, *AVF* arteriovenous fistula, *AVG* arteriovenous graft, *PS* propensity score, *OAD* oral antidiabetic drugs, *TZD* thiazolidinediones, *DPP-4 inhibitors* dipeptidyl peptidase 4 inhibitors, *ACEI / ARB* angiotensin converting enzyme inhibitors/angiotensin II receptor blockers, *ESA* erythropoiesis-stimulating agents

^a^Standardized difference (*d*): statistically significantly different between two comparison groups if *d* > 0.10

^*b*^Antiplatelets included aspirin, clopidogrel, cilostazol, dipyridamole and ticlopidine

### Exposure of oral activated vitamin D and landmark design

Records of oral activated vitamin D, including calcitriol and alfacalcidol, during each hemodialysis session, ambulatory care, and hospital admission were collected. Considering the relatively late initiation of activated vitamin D in uremic patients in Taiwan and high mortality rate especially in the first year of dialysis initiation, we chose the 360th day after cohort entry as the landmark time in order to obtain more patients prescribed vitamin D (180th day as an alternative in the sensitivity analysis) to recruit as many patients in the analysis as possible [32, 33]. Patients were classified as vitamin D users or non-users according to whether they were prescribed vitamin D before the landmark time, regardless of subsequent changes in vitamin D status [34]. Patients who died or were lost to follow-up before the landmark date were excluded. This study design helps to eliminate immortal time bias or “time-to-treatment” bias.

### Statistical analyses

For baseline characteristics, we used standardized difference (*d*) to compare the difference between vitamin D users and non-users, where less than 0.10 indicates a negligible difference between treatment groups [35, 36].

We reported crude mortality rate and estimated overall survival using Kaplan-Meier method. Conditional landmark analysis with Cox proportional hazards regression was used to evaluate mortality hazard ratios (HR) in relation to activated vitamin D use, adjusting for potential confounders. The covariates of the model included age, sex, vascular access type, baseline comorbidities, and medications.

All statistical analyses were conducted using SAS version 9.4 (SAS Institute Inc., Cary, NC).

### Propensity score method

To minimize potential confounding, we calculated propensity score (PS) of oral activated vitamin D prescriptions using age, sex, vascular access type, baseline comorbidities, and co-medications. PS trimming and inverse probability treatment weighting (IPTW) were applied to estimate population average treatment effects. Greedy algorithm was employed to match vitamin D users to non-users on PS with a ratio of 1:3 [37]. Mortality hazard ratio was estimated using PS trimming, IPTW weighting, and PS matching.

### Trajectory model

To examine the dose gradient between vitamin D use and clinical outcomes, we calculated cumulative dosage in three 120-day periods within the first 360 days of hemodialysis initiation. Only those who survived 360 days were included in the analysis. In dialysis patients, the initiation and titration dosage of calcitriol or alfacalcidol are mostly 0.25 μg per day or every other day [38, 39]. We thus defined 0.25 μg as the single dosage unit for activated vitamin D for ease of reference.

For the dynamic nature of vitamin D prescription over time, we modeled the three 120-day cumulative dosage as the longitudinal outcome and used logistic regression for the group-based trajectory models [40]. Patients were classified into high-dose and average-dose users. We evaluated where the dose-response relationship existed.

### Sensitivity analyses

Two sensitivity analyses were performed. It has been noted that a high incidence of drug record discrepancies existed in out-patient hemodialysis [41]. One of the most common medication-related problems is “indication without drug therapy” [42, 43]. To solve this, we performed the first sensitivity analysis by analyzing patients who received maintenance hemodialysis in hospital-based dialysis units from the 345th through 375th day of hemodialysis initiation. The urbanization of city/township where the hospital was located and the hospital accreditation level were incorporated into the Cox and PS models [44].

Using the landmark design, the patient selection was conditioned on the survival time [34]. Based on the study of the primary analysis, we included patients who survived more than 360 days to ensure adequate observation periods for vitamin D observation. However, the design limited the generalizability of our finding. We performed the second sensitivity analysis by change the landmark time to the 180th day of cohort entry to justify the robustness of our finding.

## Results

**Fig. 1:**
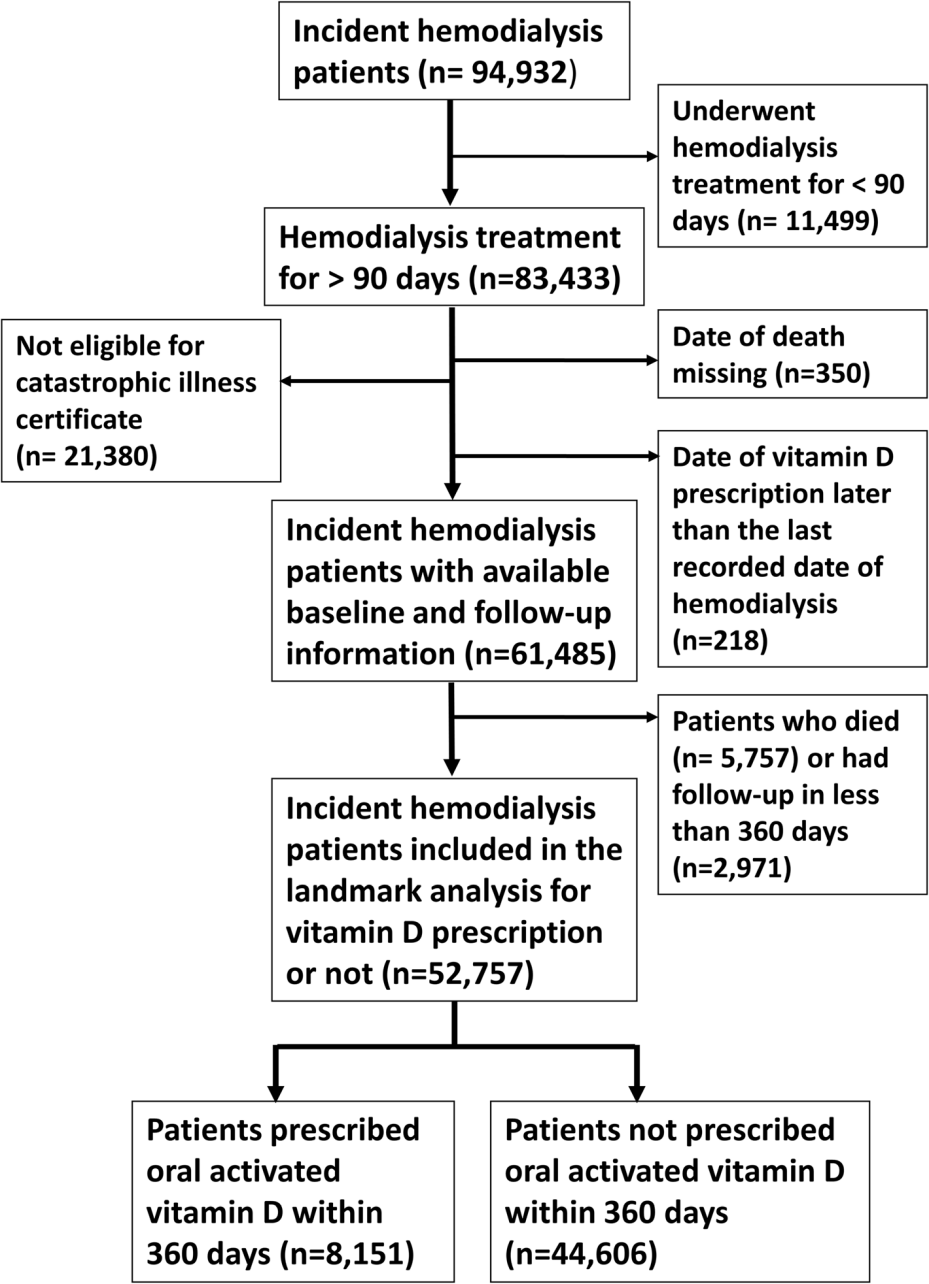
Flow diagram shows inclusion of hemodialysis patients for analysis. Numbers of incident hemodialysis patients included for analysis, linked to the outpatient and admission claims from the catastrophic illness certificate (CIC) for end-stage renal disease (ESRD) database within the National Health Insurance Research Database (NHIRD)

Between Jan 1, 2001 and June 30, 2009, there were 83,433 incident uremic patients who had undergone hemodialysis treatment for more than 90 days. After exclusion of those who were not eligible for CIC (*n* = 21,380) either due to renal function recovery or non-continuation of dialysis therapy, those registered “dead” but with missing death date (*n* = 350), and those with date of vitamin D prescription later than the last recorded date of dialysis therapy (*n* = 218), a total of 61,485 patients were included (Fig. 1). Patients who were not eligible for CIC were healthier and had fewer comorbidities (data not shown).

Of these 61,485 patients, 15,793 (25.7%) patients had ever been prescribed oral activated vitamin D during the follow-up period. The median duration of vitamin D use were 354 days (IQR 89–973 days). Among these patients, 8867 (56.1%) received vitamin D in the first 360 days after hemodialysis initiation (Additional file 1: Table S1).

Patients who died (*n* = 5757) or had follow-up less than 360 days (*n* = 2971) were excluded from analysis (Fig. 1). Vitamin D users (*n* = 8151) were significantly younger and healthier than non-users (*n* = 44,606), with less prevalence of diabetes and accompanying past histories of myocardial infarction or stroke. Vitamin D users also had more prevalent use of arteriovenous fistula and less use of graft or permanent catheters as long-term vascular access (Table 1).

**Fig. 2:**
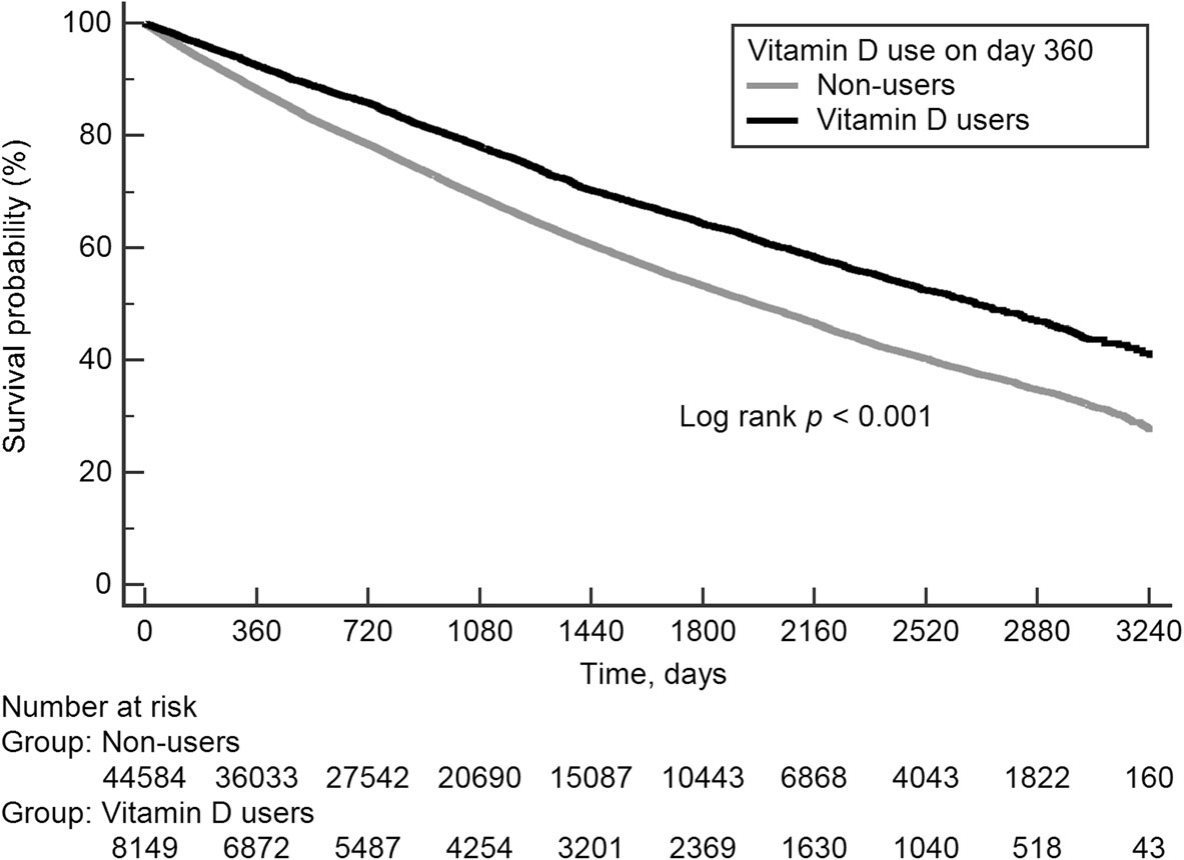
Kaplan-Meier Survival curve of activated vitamin D users versus non-users according to status by landmark time. Vitamin D users had a significantly lower risk of death, compared with non-users. Note: The landmark time is the 360th day of initiation of hemodialysis

By the end of the follow-up from the landmark time (median 1019, IQR 473–1777 days), there were 2619 deaths during 29,158.6 person-years of observation (crude mortality rate 8.98 per 100 person-years) among vitamin D users, as compared with 18,482 deaths during 142,948.7 person-years follow-up (12.93 per 100 person-years) among non-users (Additional file 1: Table S2). The survival curve of activated vitamin D users and non-users was shown (Fig. 2). Vitamin D users were less likely to die compared to non-users in unadjusted (HR 0.69 [95% CI, 0.66–0.72]) and multivariate adjusted model (HR 0.91 [95% CI, 0.87–0.95]) (Table 2).

**Table 2:** Multivariate Cox proportional hazards models examining activated vitamin D treatment as compared with no treatment by landmark time

Model	HR (95% CI)
Unadjusted	0.69 (0.66–0.72)
Adjusted	
Age and sex	0.79 (0.76–0.82)
Age, sex, and comorbidities	0.90 (0.86–0.94)
Age, sex, vascular access type, and comorbidities	0.90 (0.87–0.94)
Age, sex, comorbidities, and medications	0.90 (0.87–0.94)
Age, sex, vascular access type, comorbidities and medications	0.91 (0.87–0.95)
Propensity score (PS) method	
PS trimming (1–99%)	0.71 (0.68–0.74)
PS trimming + IPTW	0.94 (0.92–0.96)
PS matching	0.94 (0.90–0.98)

*Note*: The landmark time is the 360th day of initiation of hemodialysis

Propensity score (PS): PS was calculated with logistic regression using covariates of age, sex, vascular access type, baseline comorbidities, and medications. The PS matched methods we employed compared vitamin D users versus non-users without further adjustment of baseline covariates

*Abbreviation*: *HR* hazard ratio, *CI* confidence intervals, *PS* propensity score, *IPTW* inverse probability treatment weighting

After propensity score method employed and matching, the baseline covariates were balanced between vitamin D users and non-users (Table 1). The overlap of the distribution of propensity score across vitamin D users and non-users were displayed, before and after PS matching (Additional file 1: Figures S1 and S2), respectively. Vitamin D users still had a lower risk of death with the method of PS trimming (HR 0.71 [95% CI, 0.68–0.74]), IPTW (HR 0.94 [95% CI, 0.92–0.96]), and PS matching (HR 0.94 [95% CI, 0.90–0.98]) (Table 2). We had further performed a matched pairs analysis from which vitamin D users still had a lower risk of death (HR 0.91 [95% CI, 0.86–0.96]), compared with non-users.

To evaluate prescribing pattern and examine the dose response relationship, ambulatory claims for activated vitamin D prescriptions were collected in the first 360 days after hemodialysis initiation. Using 0.25 μg as dosage unit, the median (IQR) cumulative dosage were 80 (35–168), 60 (30–112) and 60 (30–112) units in three 120-day intervals, respectively (Additional file 1: Table S3).

**Fig. 3:**
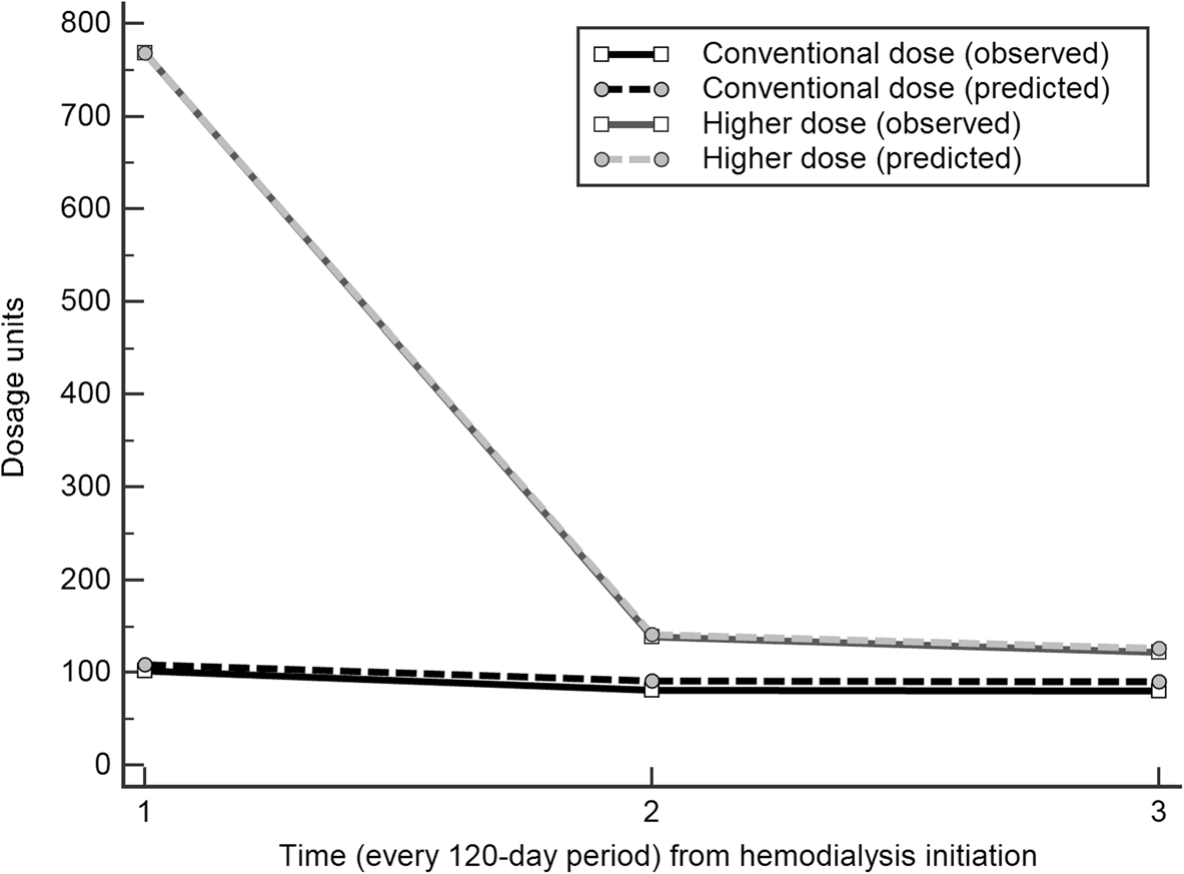
Result of group-based trajectory analysis. Trajectory of vitamin D dosage grouping from initiation of hemodialysis in the first 360 days. Trajectory model using 2 groups. Every 0.25 μg of calcitriol or alfacalcidol was defined as one dosage unit. The predicted dosage unit in each group is plotted with dotted lines. The observed proportion of individuals in each group are plotted in solid lines. After exclusion of the patients with upper 99th percentile dosage (*n* = 196) and application of trajectory analysis, the majority (dark black line) of vitamin D users (*n* = 6849) received a median of 110 (IQR 45–220) dosage units, while the remaining 326 patients (grey line) received higher cumulative dosages, median 805 (IQR 635–1080) dosage units, in the first 360 days

In the trajectory analysis (Additional file 1: Appendix S4), 326 (6.2%) patients were noted to have been given higher than average doses, while the remaining 6849 (93.8%) were prescribed the conventional daily dosage (Fig. 3). Whether high dose or conventional dose vitamin D users, they were prescribed higher dose in the first 120 days. After adjustments of potential confounders, we observed a significant survival benefit in patients receiving conventional dose (HR 0.88 [95% CI, 0.84–0.92]) and high dose activated vitamin D (HR 0.66 [95% CI, 0.55–0.78]) (Table 3). Compared with conventional dosage group, the high dose group still had a lower risk of death (HR 0.75 [95% CI, 0.63–0.89]).

**Table 3:** Crude mortality rate and multivariate adjusted hazard ratio for mortality according to the different dosage categories of oral activated vitamin D based on trajectory analysis

	N (%)	Follow-up (person-years)	Death (%)	Crude mortality rate (per 100 person-year)	Adjusted HR^b^ (95% CI)
Non-users	45,386 (86.0)	145,396.7	18,853 (41.5)	12.97	Reference
Conventional dose vitamin D	6849 (13.0)	24,398.0	2112 (30.8)	8.66	0.88 (0.84–0.92)
High dose vitamin D users^a^	522 (1.0)	2312.6	136 (26.1)	5.88	0.66 (0.55–0.78)
Overall	52,757 (100)	172,107.3	21,101 (40.0)	12.26	

*Abbreviations*: *HR* hazard ratio, *CI* confidence intervals

^a^The high dose vitamin D users consisted of the upper 99th percentile of dosage prescriptions (*n* = 196) that were previously excluded from trajectory analysis plus the minority of higher dose vitamin D users (*n* = 326) in the trajectory analysis

^b^The Cox model was adjusted by covariates including age, sex, vascular access type, baseline comorbidities, and medications

We did sensitivity analyses by analyzing patients who had regular hemodialysis in hospital-based dialysis units. The activated vitamin D users (*n* = 5449) were still younger (58.7 versus 62.1 years) and had fewer baseline comorbidities than non-users (*n* = 23,245). The crude mortality rate was lower in vitamin D users compared with non-users (8.60 versus 12.36 per 100 person-years) (Additional file 1: Table S2). After adjustment for age, sex, vascular access type, comorbidities, medications, urbanization, and hospital levels, vitamin D users were still associated with a lower risk of death (HR 0.91 [95% CI, 0.87–0.96]). Using PS matching, vitamin D users still had a lower risk of death (HR 0.95 [95% CI, 0.89–1.00]) (Table 4).

**Table 4:** Multivariate Cox proportional hazard models examining activated vitamin D treatment as compared with no treatment by landmark time in hospital-setting hemodialysis patients

Model	HR (95% CI)
Unadjusted	0.69 (0.66–0.73)
Adjusted	
Urbanization and hospital level	0.72 (0.68–0.76)
Age and sex	0.78 (0.74–0.82)
Age, sex, urbanization, and hospital level	0.80 (0.76–0.84)
Age, sex, vascular access type, and comorbidities	0.89 (0.85–0.94)
Age, sex, comorbidities, and baseline medications	0.90 (0.85–0.95)
Age, sex, urbanization, hospital level, vascular access, comorbidities, and baseline medications	0.91 (0.87–0.96)
Propensity score (PS) method	
PS trimming (1–99%)	0.70 (0.67–0.74)
PS trimming + IPTW	0.95 (0.92–0.97)
PS matching (1: 3)	0.95 (0.89–1.00)

Propensity score (PS): PS was calculated with logistic regression using covariates of age, sex, vascular access type, baseline comorbidities, medications, and levels of hospital and urbanization. The PS matched methods was employed compared vitamin D users versus non-users without further adjustment of baseline covariates

*Abbreviations*: *HR* hazard ratio, *CI* confidence intervals, *PS* propensity score, *IPTW* inverse probability treatment weighting

Additionally, we compared 6848 vitamin D users with 50,921 non-users, using the 180th day after hemodialysis initiation as the landmark time. Vitamin D users were noted to have a lower risk of death in the multivariate adjusted (HR 0.87 [95% CI, 0.84–0.91]) and PS matched model (HR 0.94 [95% CI, 0.90–0.98]), compared with non-users. After trajectory analysis and adjustment of potential confounders, high-dose vitamin D users still had a lower risk of death, compared with non-users (HR 0.64 [95% CI, 0.55–0.74]) and conventional dose users (HR 0.76 [95% CI, 0.65–0.89]), respectively.

## Discussion

In this cohort of 61,485 incident hemodialysis patients between 2001 and 2010, patients treated with oral activated vitamin D in the first 360 days after dialysis initiation had a survival advantage compared with those not treated, even after adjustment for potential confounders. The result was significant in the entire cohort using a different landmark time and subgroup of hospital-based hemodialysis patients. The presence of dose-response relationship further supported the potential benefit of activated vitamin D prescription in these patients.

According to the Dialysis Outcomes and Practice Pattern Study (DOPPS), intravenous vitamin D was most common in the United States but oral administration was more prevalent in all other countries. The percentage of patients on vitamin D were 33% in France, 66% in the United States, and 39% in Japan in the DOPPS III (2005–2006) [27]. In the Current Management of Secondary hyperparathyroidism – a multicenter Observational Study (COSMOS), 48% of prevalent hemodialysis patients in Europe were using activated vitamin D, mostly calcitriol and alfacalcidol [45].

In Taiwan, oral route but not intravenous administration of activated vitamin D is reimbursed by the NHI. In our study, we found that only 25.7% of patients had ever been prescribed activated vitamin D, exclusively in oral form. The prescription of activated vitamin D in Taiwan was not as prevalent or as early as those in the United States and European countries [24, 27, 45]. This may result from the different indications between vitamin D supplementation and suppression of parathyroid hyperplasia [46]. Higher geographic latitude or dark skin may be associated with a higher prevalence of vitamin D deficiency, higher PTH levels, and more prescriptions of activated vitamin D [47]. In Taiwan, the widespread use of inexpensive calcium-containing phosphate binders may lead to reduced PTH levels, which contributed to fewer prescriptions of vitamin D. In addition, the level of vitamin D was rarely tested in ESRD patients in Taiwan and activated vitamin D was often prescribed for secondary hyperparathyroidism, which often developed in the later dialysis vintage. The median time to the first prescription was 252 (IQR 31–919) days after hemodialysis initiation, obviously later than that in the DOPPS, although the exact indications and levels of PTH were not available from the NHIRD.

In the literature, oral calcitriol use was associated with lower all-cause mortality in CKD stage 3–4 patients. In these non-dialyzed CKD studies, patients given calcitriol were older, having higher PTH level and lower glomerular filtration rate, and more were diabetics [21, 23]. In contrast, evidence from observational studies of hemodialysis patients have shown that patients prescribed activated vitamin D were younger and healthier [22, 24]. Different from the above studies, our study did not choose time-dependent exposure to assess vitamin D effect because the concept of time-dependent has been thought of as more focused on the “state of exposure” on the outcome rather than the effect of early vitamin D supplement or exposure on the long-term outcome. We also did not use marginal structural model (MSM) to deal with time-varying covariates because of lack of laboratory data and detailed comorbidity information in claims data of hemodialysis treatment in the NHI. Instead, we retrieved not only diagnostic codes but comprehensive medication use and vascular access type obtained from claims data of all medical services during baseline periods, which were deemed reliable for input in PS to adjust for imbalance between vitamin D users versus non-users.

Survival benefits of oral calcitriol have been found, in those receiving mean daily doses of less than 1 μg [22]. However, the author also found that the lower the vitamin D dose, the lower the risk of death. Using MSM, Miller et al. [48]. have found that higher dose paricalcitol was associated with greater survival in hemodialysis patients but failed to confirm this using conventional Cox model or PS matched method. However, patients taking paricalcitol represented a small proportion of the hemodialysis population in the U.S., and thus, the result could not be extrapolated to populations in other countries [48]. Concerning the high cost, paricalcitol is not reimbursed in the NHI and thus rarely used in Taiwan practically.

Randomized controlled trials comparing activated vitamin D use versus placebo are unacceptable ethically. Thus, observational studies still have a role in leading the trend of clinical practice.

The strength of this study is the large real-life cohort with detailed information of comorbidities and co-medications and a long follow-up duration up to 10 years. In addition, the inclusion of incident hemodialysis patients with utilization of landmark design reduced immortal time bias [49]. Although the design of landmark analysis introduced misclassification bias when some vitamin D users were categorized into non-users, as may underestimate the effect of vitamin D, the true beneficial effect must be even greater since we found a lower risk of mortality in vitamin D users. Despite lack of active comparators, we adopted PS matching and reduced the imbalance between users and non-users.

Additionally, our study had illustrated trajectories of vitamin D prescription dosage and to highlighted the temporal changes in the first 360 days of dialysis initiation. It is straightforward to use trajectories to classify different dosage groups which may help us to determine the dose exposure patterns. The positive association of higher dose calcitriol or alfacalcidol and reduced all-cause mortality in our analysis further supported the beneficial effect of activated vitamin D in hemodialysis patients. Reducing use of calcium-based phosphate binders should be considered to trade off for more activated vitamin D prescriptions to avoid the risk of hypercalcemia, inadequately suppressed PTH levels, or low bone turnover disease. Further study may be needed.

One major limitation of our study is that there were no laboratory data such as calcium, phosphorus, PTH, hemoglobin, smoking status, and markers of inflammatory status available from Taiwan NHI medical claims.

We conducted a stratified analysis in only female patients to minimize the potential confounding by smoking since the prevalence of smoking is very low (4.3%) among female population in Taiwan [50]. Compared with non-users, vitamin D users were associated with a lower all-cause mortality risk (HR 0.89 [95% CI, 0.84–0.94]) in females who were largely non-smokers. Such reduced effect observed in females was also similarly observed in male patients (HR 0.93 [95% CI, 0.87–0.98]), who had a smoking prevalence of 46.8%. This sex-stratified analyses provided further reassurance that the potential of confounding by smoking is very small in our study.

The overall mortality in this hemodialysis cohort in Taiwan was substantially lower than that in other countries, as may result from different race, life style, or fewer cardiovascular events and better medical accessibility due to comprehensive health insurance coverage [30, 51]. The observation from our study implies that using inexpensive activated vitamin D may bring about significant survival benefit, even though newer vitamin D analogs with fewer hypercalcemic side effects were not prescribed extensively in Taiwan.

## Conclusions

In incident hemodialysis patients, treatment of oral calcitriol or alfacalcidol was associated with lower risks of death. There was no excess risk for death in patients receiving higher doses of vitamin D. Therefore, our data supports the prescription of activated vitamin D in these patients unless contraindicated.

## Additional file

Additional file 1: **Table S1.** The frequency in incident hemodialysis patients according to first-time prescription of activated vitamin D. **Table S2.** Events of death and crude mortality rates by status of vitamin D use on the landmark time in the entire cohort and subgroup of patients in hospital-based hemodialysis setting. **Table S3.** Cumulative and average dosage units of vitamin D use in each 120-day period of the first 360 days of hemodialysis initiation. **Appendix S1.** Details of diagnostic codes to retrieve comorbidity information from baseline period. **Appendix S2.** Details of prescribed medication during baseline period. **Appendix S3.** Details of procedure codes of vascular access type. **Appendix S4.** Details of trajectory model for vitamin D dosage category. **Figures S1 and S2.** The distribution of propensity score across vitamin D users and non-users before and after propensity score matching (DOCX 86 kb)

## Abbreviations

ACEI / ARB: angiotensin converting enzyme inhibitors/angiotensin II receptor blockers
AVF: Arteriovenous fistula
AVG: Arteriovenous graft
CHF: Congestive heart failure
Chronic liver diseases: Chronic viral hepatitis, cirrhosis and its complications
CIC: Catastrophic illness certificate
CKD: Chronic kidney disease
COPD: Chronic obstructive pulmonary disease
CTD: Connective tissue disease including rheumatoid arthritis, systemic lupus erythematosus, etc.
CVD: Cerebrovascular disease
DM: Diabetes mellitus
DPP-4 inhibitors: Dipeptidyl peptidase 4 inhibitors
ESA: Erythropoiesis-stimulating agents
ESRD: End stage renal disease
IPTW: Inverse probability treatment weighting
MI: Myocardial infarction
NHI: National Health Insurance
NHIRD: National Health Insurance Research Database
OAD: Oral antidiabetic drugs
PS: Propensity score
PTH: Parathyroid hormone
PUD: Peptic ulcer disease
PVD: Peripheral vascular disease
TZD: Thiazolidinediones
