# Supplementary material for: Assessing the effect of oral activated vitamin D on overall survival in hemodialysis patients: a landmark analysis
Source: BMC Nephrol. 2018 Nov 6;19:309. doi: 10.1186/s12882-018-1111-2 (PMC6219061; doi:10.1186/s12882-018-1111-2)
Supplement: Supplementary file 1 — Table S1. The frequency in incident hemodialysis patients according to first-time prescription of activated vitamin D. Table S2. Events of death and crude mortality rates by status of vitamin D use on the landmark time in the entire cohort and subgroup of patients in hospital-based hemodialysis setting. Table S3. Cumulative and average dosage units of vitamin D use in each 120-day period of the first 360 days of hemodialysis initiation. Appendix S1. Details of diagnostic codes to retrieve comorbidity information from baseline period. Appendix S2. Details of prescribed medication during baseline period. Appendix S3. Details of procedure codes of vascular access type. Appendix S4. Details of trajectory model for vitamin D dosage category. Figures S1 and S2. The distribution of propensity score across vitamin D users and non-users before and after propensity score matching (DOCX 86 kb) [file 12882_2018_1111_MOESM1_ESM.docx]

**Supporting information**

**Assessing the Effect of Oral Activated Vitamin D on Overall Survival in Hemodialysis Patients**

Jo-Yen Chao, MD,^1,2^ Hsu-Chih Chien, BSPharm, PhD,^2^ Te-Hui Kuo, MD,^1,3^ Yu-Tzu Chang, MD, PhD,^1,4^ Chung-Yi Li, PhD,^3^ Ming-Cheng Wang, MD^1,2^, Yea-Huei Kao Yang, BSPharm^2^

^1^Division of Nephrology, Department of Internal Medicine, National Cheng Kung University Hospital, College of Medicine, National Cheng Kung University, Tainan, Taiwan

^2^Institute of Clinical Pharmacy and Pharmaceutical Sciences, College of Medicine, National Cheng Kung University, Tainan, Taiwan

^3^Department of Public Health, College of Medicine, National Cheng Kung University, Tainan, Taiwan

^4^Graduate Institute of Clinical Medicine, College of Medicine, National Cheng Kung University, Tainan, Taiwan

**S1 Table.** **The frequency in incident hemodialysis patients according to first-time prescription of activated vitamin D**

**S2 Table. Events of death and crude mortality rates by status of vitamin D use on the landmark time in the entire cohort and subgroup of patients in hospital-based hemodialysis setting**

**S3 Table**. **Cumulative and average dosage units of vitamin D use in each 120-day period of the first 360 days of hemodialysis initiation**

**S1 Appendix**. **Details of diagnostic codes to retrieve comorbidity information from baseline period.**

**S2 Appendix**. **Details of prescribed medication during baseline period.**

**S3 Appendix. Details of procedure codes of vascular access type.**

**S4 Appendix. Details of trajectory model for vitamin D dosage category**

**S1 and S2 Figures. The distribution of propensity score across vitamin D users and non-users before and after propensity score matching**

**Table S1. The frequency in incident hemodialysis patients according to first-time prescription of activated vitamin D**

| **First time of vitamin D prescription** | **N (%)** | **Cumulative percent** |
| --- | --- | --- |
| **≤ 90 days** | 5,588 (35.4) | 35.4 |
| **> 90 and ≤ 180 days** | 1,503 (9.5) | 44.9 |
| **> 180 and ≤ 360 days** | 1,776 (11.3) | 56.2 |
| **> 360 and ≤ 720 days** | 2,140 (13.6) | 69.8 |
| **> 720 and ≤ 1080 days** | 1,465 (9.3) | 78.9 |
| **> 1080 days** | 3,324 (21.1) | 100.0 |
| **Total** | 15,793 (100.0) |  |

**Table S2. Events of death and crude mortality rates by status of vitamin D use on the landmark time in the entire cohort and subgroup of patients in hospital-based hemodialysis setting**

| ***Primary analysis*** | **N (%)** | **Follow-up (person-years)** | **Death (%)** | **Crude mortality rate**  **(per 100 person-years)** |
| --- | --- | --- | --- | --- |
| **Landmark time on the 360^th^ day** |  |  |  |  |
| Vitamin D users | 8,151 (15.5) | 29,158.6 | 2,619 (32.1) | 8.98 |
| Non-users | 44,606 (84.5) | 142,948.7 | 18,482 (41.4) | 12.93 |
| Overall | 52,757 (100.0) | 172,107.3 | 21,101 (40.0) | 12.16 |
| ***Sensitivity analyses*** |  |  |  |  |
| **Hospital-setting hemodialysis patient** | | | | |
| Vitamin D users | 5,449 (19.0) | 18,882.5 | 1,623 (29.5) | 8.60 |
| Non-users | 23,245 (81.0) | 74,982.7 | 9,271 (39.9) | 12.36 |
| Overall | 28,694 (100.0) | 93,865.2 | 10,894 (38.0) | 11.61 |
| **Landmark time on the 180^th^ day** |  |  |  |  |
| Vitamin D users | 6,848 (11.9) | 26,789.9 | 2,328 (33.9) | 8.69 |
| Non-users | 50,921 (88.1) | 172,465.4 | 22,451 (44.1) | 13.02 |
| Overall | 57,769 (100.0) | 199,255.3 | 24,779 (42.9) | 12.44 |

**Appendix S1. Details of diagnostic codes to retrieve comorbidity information from baseline period.**

| Comorbidities | ICD-9 code |
| --- | --- |
| Diabetes mellitus | 250 |
| Congestive heart failure | 398.91, 402.01, 402.11, 402.91, 404.01, 404.03, 404.11, 404.13, 404.91, 404.93, 425.4, 425.5 - 425.9, 428 |
| Myocardial infarction | 410, 411, 412, 413, 414 |
| Peripheral vascular disease | 437.3, 440, 441, 443.1 – 443.9, 447.1, 557.1, 557.9, V434, 093 |
| Cerebrovascular disease | 431, 433, 434, 435, 436, 437, 438 |
| Chronic lung disease | 416.8, 416.9, 490.0 - 505.9, 506.4, 508.1, 508.8 |
| Connective tissue disease | 446, 714, 720, 725, 701.0, 710.0 - 710.4, 710.8, 710.9, 711.2, 719.3, 728.5, 728.89, 729.3 |
| Peptic ulcer disease | 531.7, 531.9, 532.7, 532.9, 533.7, 533.9, 534.7, 534.9 |
| Neoplasia | 140 - 172, 174 - 195, 200 - 202, 203, 205 - 208, 238.6 |
| Chronic liver diseases | 070.22, 070.23, 070.32, 070.33, 070.44, 070.54, 070.6, 456.0 - 456.2, 571.2, 571.3, 571.5 - 571.9, 572.2 - 572.8, 573.4, V427, 570 |

From the claims data of ambulatory care or hospital admission within 90 days prior to or after the date of cohort entry, i.e. the baseline period, we applied the diagnostic codes modified from Elixhauser comorbidity index to define baseline comorbidities.

**Appendix S2 Details of prescribed medication during baseline period.**

|  | Medication and ATC code |
| --- | --- |
| Antiplatelets | Clopidogrel (B01AC04), ticlopidine (B01AC05), aspirin (B01AC06), dipyridamole (B01AC07), cilostazol (B01AC23) |
| Anticoagulant | warfarin (B01AA03) |
| Statins | Simvastatin (C10AA01), lovastatin (C10AA02), pravastatin (C10AA03), fluvastatin (C10AA04), atorvastatin (C10AA05), rosuvastatin (C10AA07), pitavastatin (C10AA08) |
| Insulin | Human insulin (A10AB01, A10AC01), insulin aspart (A10AB05), insulin glulisine (A10AB06), insulin glargine (A10AE04), insulin detemir (A10AE05) |
| Oral antidiabetic agents | |
| Biguanides | Metformin (A10BA02) |
| Sulfonylurea | Glibenclamide (A10BB01), chlorpropamide (A10BB02), tolbutamide (A10BB03), glibornuride (A10BB04), tolazamide (A10BB05), glipizide (A10BB07), gliquidone (A10BB08), gliclazide (A10BB09), glimepiride (A10BB12), acetohexamide (A10BB31) |
| Alpha-glucosidase inhibitors | Acarbose (A10BF01), miglitol (A10BF02) |
| Thiazolidinediones | Rosiglitazone (A10BG02), pioglitazone (A10BG03) |
| Dipeptidyl peptidase 4 (DPP-4) inhibitors | Sitagliptin (A10BH01), vildagliptin (A10BH02), saxagliptin (A10BH03), alogliptin (A10BH04), linagliptin (A10BH05) |
| Meglitinides | repaglinide (A10BX02), nateglinide (A10BX03) |
| ACEIs | Captopril (C09AA01), enalapril (C09AA02), lisinopril (C09AA03), perindopril (C09AA04), ramipril (C09AA05), quinapril (C09AA06), benazepril (C09AA07), cilazapril (C09AA08), fosinopril (C09AA09), imidapril (C09AA16) |
| ARBs | Losartan (C09CA01), eprosartan (C09CA02), valsartan (C09CA03), irbesartan (C09CA04), candesartan (C09CA06), telmisartan (C09CA07), olmesartan medoxomil (C09CA08), azilsartan medoxomil (C09CA09) |
| Beta-blockers | Alprenolol (C07AA01), oxprenolol (C07AA02), pindolol (C07AA03), propranolol (C07AA05), timolol (C07AA06), sotalol (C07AA07), nadolol (C07AA12), carteolol (C07AA15), metoprolol (C07AB02), atenolol (C07AB03), acebutolol (C07AB04), betaxolol (C07AB05), bisoprolol (C07AB07), esmolol (C07AB09), labetalol (C07AG01), carvedilol (C07AG02) |
| Diuretics | Bendroflumethiazide (C03AA01), hydroflumethiazide (C03AA02), hydrochlorothiazide (C03AA03), trichlormethiazide (C03AA06), cyclopenthiazide (C03AA07), furosemide (C03CA01), bumetanide (C03CA02), spironolactone (C03DA01), eplerenone (C03DA04), amiloride (C03DB01) |
| Erythropoietin stimulating agents | Erythropoietin (B03XA01), darbepoietin alfa (B03XA02), methoxy polyethylene glycol-epoietin beta (B03XA03) |

**ATC: The WHO Anatomical Therapeutic Chemical classification system**

From the claims data of ambulatory care or hospital admission within 90 days prior to or after the date of cohort entry, i.e. the baseline period, we retrieved the prescriptions of above medication to define baseline medication use.

**Appendix S3. Details of procedure codes of vascular access type.**

| **Vascular access type** | **NHI procedure code** | **NHI procedure code description** |
| --- | --- | --- |
| **AVF** | 69032B, 69032C | Repair and anastomosis of peripheral vessel |
| **AVG** | 69032BC, 69034C, | A-V shunt with Gore-tex graft |
|  | 69038C | Repair or anastomosis of peripheral vessel with graft |
| **Permanent catheter** | 47059B | Therapeutic catheter implantation — Hickman catheter implantation |
|  | 47061B, 69039B, 37024AD | Permanent Catheter implantation through internal jugular vein |
| **Double lumen catheter** | 69006C | Insertion cannula for hemodialysis or other purpose, vein to vein |
| **Unknown** | 69005B | Exploration, vascular |

The NHI procedure codes and descriptions were quoted from the Taiwan NHI billing codes for reimbursement claims. The NHI billing codes and the associated information were cited from <https://www.nhi.gov.tw/query/query2.aspx>

**Appendix S4. Details of trajectory model for vitamin D dosage category.**

To evaluate prescribing pattern and examine the dose response relationship, ambulatory claims for activated vitamin D prescriptions were collected in the first 360 days after hemodialysis initiation. Using 0.25 μg as dosage unit, the median (IQR) cumulative dosage were 80 (35-168), 60 (30-112) and 60 (30-112) units in three 120-day intervals, respectively (**S3 Table**).

For the dynamic nature of vitamin D prescription over time, we modeled the three 120-day cumulative dosage as the longitudinal outcome and used logistic regression for the group-based trajectory models. The time variable was the order of 120-day interval since hemodialysis initiation (interval 1, 2 and 3). Patients were classified into high-dose and average-dose users.

In the trajectory analysis, we excluded the upper 99^th^ percentile dosage of vitamin D prescriptions (n=196) and included 7,175 patients in the trajectory analysis. Among these patients, 326 (6.2%) were noted to have been given higher than average doses, especially in the first 120-day period, while the remaining 6,849 (93.8%) were prescribed the conventional daily dosage in the 360-day period (**Fig 3**).

**Table S3. Cumulative and average dosage units of vitamin D use in each 120-day period of the first 360 days of hemodialysis initiation**

|  | **Phase 1**  **(0 to 120 day)** | **Phase 2**  **(121 to 240 day)** | **Phase 3**  **(241 to 360 day)** |
| --- | --- | --- | --- |
| **Vitamin D users (N)** | 4,901 | 3,949 | 3,767 |
| **Cumulative dosage units** | | | |
| **Median (Q1-Q3)** | 80 (35-168) | 60 (30-112) | 60 (30-112) |
| **Daily dosage units** | | | |
| **Mean (SD)** | 1.40 (2.70) | 0.70 (0.64) | 0.69 (0.65) |
| **Median (Q1-Q3)** | 0.67 (0.29-1.40) | 0.50 (0.25-0.93) | 0.50 (0.25-0.93) |

Ambulatory claims for activated vitamin D prescriptions were collected in the first 360 days after hemodialysis initiation. A total of 634,989 prescriptions were identified for patients who survived 360 days. Using 0.25 μg as dosage unit, the median (IQR) cumulative dosage were 80 (35-168), 60 (30-112) and 60 (30-112) units in three 120-day intervals, respectively.

**Figures S1 and S2 . The distribution of propensity score across vitamin D users and non-users before and after propensity score matching**


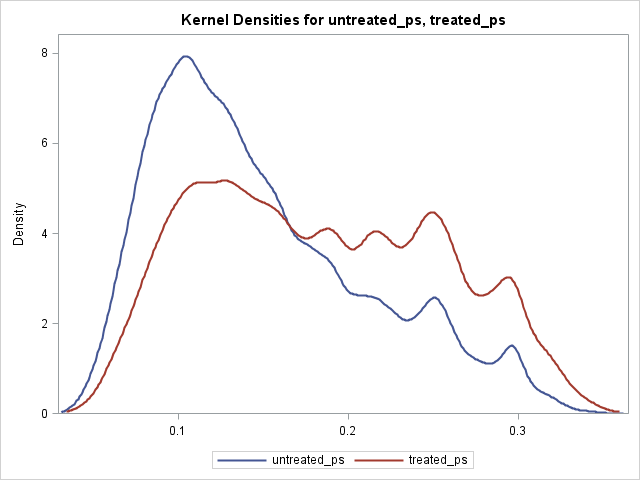


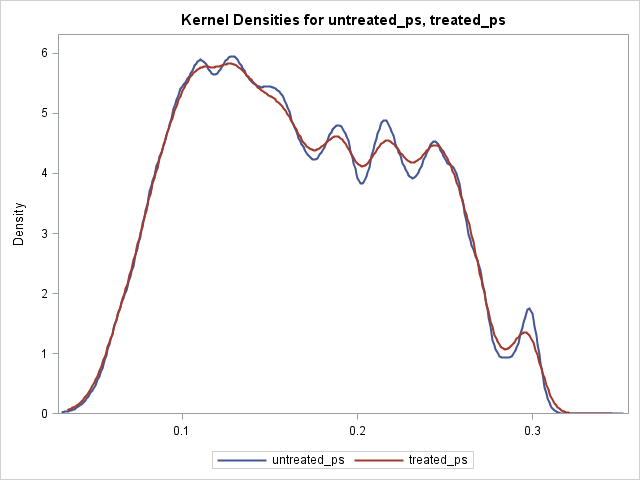


The distribution of propensity score (PS) of vitamin D treated (red color) and non-treated (blue color) before the landmark 360^th^ day is shown in the upper figure. After PS matching (the lower figure), the density plot of vitamin D user group was well-matched to non-users.
